# Efficacy and safety of combination of empagliflozin and metformin with combination of sitagliptin and metformin during Ramadan: an observational study

**DOI:** 10.1186/s12902-022-01168-3

**Published:** 2022-10-13

**Authors:** Ibrar Ahmed, Umar Yousaf Raja, Muhammad Umar Wahab, Tejhmal Rehman, Osama Ishtiaq, A. H. Aamir, Tahir Ghaffar, Abbas Raza, Suresh Kumar, Akhtar Sherin, Faisal Masood, Fawad Ahmad Randhawa, Ali Asghar, Sehrish Khan

**Affiliations:** 1 Lady Reading Hospital, Soekarno Rd, PTCL Colony, Peshawar, House No 6A, Street 2, Akbar Town Danishabad, Near Academy Hostel, Peshawar, Khyber Pakhtunkhwa Pakistan; 2 Shifa International, Pitras Bukhari Road, Sector H-8/4, Islamabad, Pakistan; 3 Umer Diabetes and Foot Clinic, Malak shafait plaza, Mauza Mahal kot, Hathial, Main Murree Rd, Bhara Kahu, Islamabad, Pakistan; 4 Hayatabad Medical Complex, Phase-4 Phase 4 Hayatabad, Peshawar, Khyber Pakhtunkhwa Pakistan; 5 Shaukat Khanum Hospital, 153-E, Shah Noor Park (adjacent Clinix Pharmacy Head Office), Main Multan Road, Lahore, Pakistan; 6 Bolan Medical, Brewery Rd, Quetta, Balochistan Pakistan; 7 KMU Institute of Medical Sciences, KIMS, Phase 2, KDA, Khyber Pakhtoonkhwa, Phase 2 Kohat Development Authority (KDA), Kohat Development Authority, KohatKohat, Khyber Pakhtunkhwa Pakistan; 8 Alkhaliq Hospital, Nishtar Rd، Al Rahim Colony, Multan, Punjab Pakistan; 9 Mayo Hospital, Hospital Rd, Anarkali Bazaar, Lahore, Punjab Pakistan; 10 Liaquat National Hospital, National Stadium Rd, Liaquat National Hospital, Karachi, Karachi City, Sindh Pakistan

**Keywords:** Diabetes, Ramadan, Fasting, Hypoglycemia

## Abstract

**Background:**

Management of diabetes during fasting is a clinical challenge. Sodium glucose co-transporter -2 inhibitors (SGLT2i) are considered safe with a low risk of hypoglycemia. However, studies on SGLT2i are scarce. This study was designed to compare the efficacy, safety, and tolerability of empagliflozin with metformin during Ramadan in comparison with sitagliptin and metformin.

**Methods:**

It was a prospective, observational study, conducted at 11 different sites all across Pakistan on an outpatient basis during Ramadan (May 2021–June 2021). including 132 patients, 88 who received metformin and sitagliptin, and 44 patients who received metformin and empagliflozin.

**Results:**

Patients of the SGLT-2i group experienced similar symptomatic hypoglycemic episodes (15.9%) as the sitagliptin group. There was an improvement in blood sugar levels after the use of SGLT-2i (RBS 181 ± 64 before Ramadan vs 162 ± 53 after Ramadan). HbA1c also improved after the use of SGLT-2i before and after Ramadan (7.2 ± 0.8 vs 6.9 ± 0.9 for Metformin + Empagliflozin and 7.8 ± 1.5 vs 7.6 ± 1.6 for Metformin and sitagliptin). Weight and BMI improved after the use of SGLT-2i (BMI 36.5 ± 4.8 before Ramadan and 33.7 ± 2.4 after Ramadan). There were no reported cases of urinary tract infection in the empagliflozin group.

**Conclusion:**

SGLT-2 inhibitors combined with metformin for patients with diabetes during Ramadan fasting is as effective, safe and well tolerated as DPP4 combined with metformin.

## Background

Ramadan is a lunar-based month lasting between 29 − 30 days. Fasting during the holy month of Ramadan constitutes one of the five main pillars of Islam and it is obligatory for all healthy adult Muslims to fast during Ramadan. The duration of fasting varies from 12 to 20 h, with the average fasting period being 15 h [1]. Although people who are ill, travelling and those who are pregnant are exempted by Islam from fasting, many persons with diabetes still choose to fast during Ramadan, even against their health care professionals advice [1, 2].

The type of medication the patient is taking for diabetes management influences the potential risks that fasting may cause and needs careful attention within the treatment plan. Adjustments to the dose and timing of some medications may be required during Ramadan to minimize the risk of hypoglycemia, hyperglycemia and dehydration in fasting patients along with Ramadan specific diabetes education. [3]

Several observational and randomized controlled studies have been conducted on people with Type 2 diabetes mellitus (T2DM) fasting during Ramadan, focusing on the safety profile of different available oral antidiabetic agents, notably assessing the incidence of hypoglycemia episodes. [4–6] These studies have shown DPP4 inhibitors and metformin to be generally safe during Ramadan and various Ramadan guidelines have recommended continuing with DPP4 inhibitors and metformin during fasting for people with T2DM who wish to fast during Ramadan. [1, 2, 7]

Sodium glucose co-transporter-2 Inhibitor (SGLT2-inhibitor) are a newer class of oral hypoglycemic agents that have shown to be effective as monotherapy and as an add-on treatment for T2DM with significant add-on benefit regarding body weight and systolic blood pressure reduction. [8, 9] However, since the introduction of the SGLT2 inhibitors, there has been some concern among physicians about safety of SGLT2 inhibitors in patients who wish to observe fasting during the Ramadan. These concerns include an increase in urinary tract and genital infections (due to glycosuria), and the potential for volume depletion, electrolyte imbalance, ketoacidosis and increased bone fracture risk [10, 11]. These effects are particularly important during the summer months and in hot regions.

### Rationale

Keeping in view the hot climate and the long fasting hours, it was need of time to determine which medications will be beneficial for fasting people with diabetes. Therefore, the present study was designed to evaluate the efficacy and safety profile of SGLT2 inhibitor empagliflozin with metformin in comparison with DPP4 inhibitor sitagliptin with metformin in terms of hypoglycemia, dehydration and glucose control in people with T2DM observing fast during Ramadan.

## Methods

### Patients and study design

This is an observational cohort study conducted at 11 different sites all across Pakistan on outpatient basis during Ramadan (May 2021–June 2021). The study included 132 patients with type 2 diabetes mellitus of ≥ 06 months duration*.* Eighty-eight patients received metformin and sitagliptin (100 mg/day sitagliptin dose), and 44 patients received metformin and empagliflozin (10 mg/day empagliflozin dose). Study participants were already taking these medications for at least six weeks prior to Ramadan.

### Sample size estimation

It was estimated using online sample size calculator, openepi version 3.01 for mean difference, after input the change in mean HbA1c 8.2±1.5 % vs. 7.4±1.4% in Ramadan. We required at least *n* = 104 samples for this study, 52 samples per group, however due to expected drop out we are taking 130 samples per group and *n*=65 for this study.



### Inclusion and exclusion criteria

Patients with HbA1c ranging from 6.5–8.5% before Ramadan and eGFR > 60 ml/min were included. Patients were excluded if they were on Insulin or had contraindications for fasting such as severe renal disease, liver diseases, unstable angina, history of diabetic ketoacidosis, hypoglycemic unawareness as per IDF diabetes and Ramadan (DAR) guidelines [3].

### Clinical and laboratory workup

A detailed history followed by clinical examination was done in all patients fulfilling the inclusion criteria. Screening visit which was done at least 5 weeks prior to the start of Ramadan, the information regarding age, gender, history of diabetes, intention to fast in the forthcoming Ramadan, and diabetes related complications were obtained. Weight, height and blood pressure was taken. Body Mass Index (BMI) and blood pressure measurements were done before and after Ramadan. Each patient was provided individualized Ramadan specific education specially focusing on dietary modifications and frequent blood glucose monitoring by glucometer at different times of the day according to DAR IDF guidelines [3]. Blood samples were collected to measure HbA_1_c, fasting blood glucose, urine DR and kidney function tests (serum creatinine & eGFR).

Patients were provided with a log book to record important daily events especially hypoglycemic symptoms, and whether they required any assistance due to symptoms of hypoglycemia, time and duration of hypoglycemia, and whether the fast was broken due to any reason. If patients experienced symptoms of hypoglycemia, they were instructed to check blood glucose level using glucometer and enter the readings in their log books along with symptoms.

The log book was maintained by the patients on a daily basis throughout Ramadan, irrespective of symptoms. In case of multiple episodes of hypoglycemia, each episode was documented separately. Furthermore, every patient was instructed to check blood glucose measurement before the evening meal three times per week.

At the end of Ramadan (i.e., study end), a follow up visit was done to obtain information regarding the fast-during Ramadan and any changes in medications dose and timing if required were done.

Safety and tolerability of these medications during Ramadan were assessed by reported adverse events entries in log book during the study period. All reported adverse events were categorized into mild, moderate or severe and relationship to study drug by the investigator. Patients were also contacted twice weekly by phone after the completion of Ramadan to find out about the occurrence of any serious side effect.

### Primary outcomes

- a**) Hypoglycemic episodes during Ramadan** were assessed by self-monitoring of the blood glucose during the day according to IDF diabetes and Ramadan (DAR) guidelines for blood glucose monitoring during Ramadan [3]^.^ The reported hypoglycemic symptoms in our study participants included palpitations, nausea, sweating, confusion, tremors, symptoms of dizziness, visual blurring, or intense hunger with or without biochemical confirmation. Blood glucose < 70 mg/dl on finger prick test was considered as hypoglycemia and it was considered severe episode if patient required assistance from another person or that resulted in seizure or loss of consciousness.
- **b) Dehydration episodes**
- This was assessed by symptoms of hypotension, orthostatic hypotension, postural dizziness, dehydration, syncope attack during fasting.
- **c) Genitourinary Infections**
- Number of participants who experienced any urinary tract or genital infections.

### Secondary outcome

This included changes in weight and BMI after Ramadan.

### Ethical issues

The study protocol was approved by the local ethical committee and all patients signed informed consent before participation.

### Statistical analysis

Results are presented as total count, percentages, mean and standard deviation. Comparison between categorical variables was done using chi-square test while numerical variables were compared using student t-test or Mann–Whitney U test. Values of weight, BMI, HbA1c and RBS were compared before and after fasting using Paired Sample T test. A *P*-value less than 0.05 was considered statistically significant. All statistical calculations were performed using SPSS 25 (IBM, USA).

## Results

The present study included 132 patients, out of which 88 received metformin and sitagliptin (Group A), and 44 patients received metformin and empagliflozin (Group B).

Comparison between the two studied groups regarding the basic data including mean age, gender, duration of diabetes, BMI, HbA1c before Ramadan, antidiabetic medications, duration of hypertension and antihypertensive medications revealed no statistically significant differences (Table 1).

**Table 1:** Baseline characteristic of studied participants

Variables	Group A (*n* = 88) Metformin + Sitagliptin	Group B (*n* = 44) Metformin + Empagliflozin	*P* value*
Age (years) mean ± SD	50.6 ± 10.5	44.7 ± 10.7	0.027*
Variables			*P* value**
Male / Female	43 / 45 = 48.8%	24 / 20 = 54.5%	0.538**
Hypertension	37 = 42%	16 = 36.3%	
Anti HTN medications			*P* value**
Angiotensin-converting enzyme inhibitors	11 = 12.5%	2 = 4.5%	0.148
Calcium Channel Blocker	09 = 10.2%	07 = 15.9%	0.346
Angiotensin II receptor blockers	18 = 20.4%	14 = 31.8%	0.151

^*^P value calculated using Independent sample *T* test, keeping *P* value ≤ 0.5 as significant

^**^P value calculated using Pearson Sq. Test, keeping *P* value ≤ 0.5 as significant

### Safety profile between Metformin + Sitagliptin and Metformin + Empagliflozin

This was assessed by comparing the number of hypoglycemic episodes, number of days patients fasted and adverse reactions between the two groups:

- ∙ **Hypoglycemic Episodes:** Patients of SGLT-2i group experienced similar hypoglycaemic episodes (15.9%) as sitagliptin group.
- ∙ **No. of days patients fasted:** There were differences between the two groups regarding the number of days patient fasted (23 ± 12 vs 18 ± 13 days).
- ∙ Similarly comparable symptomatic hypoglycemic (15.9%, *p* value 1.0) and hyperglycemic episodes (6.8%, *p* value 1.0) were observed in both groups.
- ∙ **Adverse reactions:** Frequency of symptomatic urinary tract infections was also similar in both groups. However, there were no significant differences between the studied groups regarding the frequency of patients with episodes of volume depletion (Table 2).

**Table 2:** Adverse effects during Ramadan

Adverse Effects	Groups						*p*-value
	**Total**		**Metformin + Sitagliptin**		**Metformin + Empagliflozin**		
	**n**	**%**	**n**	**%**	**n**	**%**	
**Diarrhea**	10	7.5	08	9.1	02	4.5	0.35
**Rash**	05	3.7	05	5.7	0	0	0.10
**Hyperglycemia**	09	6.8	06	6.8	03	6.8	1.0
**Hypoglycemia**	21	15.9	14	15.9	07	15.9	1.0
**Urinary tract infection**	10	7.5	06	6.8	00	0	0.077
**Increased urination**	23	17.4	13	14.8	10	22.7	0.25
**Abdominal Pain**	12	9.0	07	8	05	11.4	0.52

*p* < 0.05 was considered significant using Independent Sample *T* Test

### Efficacy profile between metformin + sitagliptin and metformin + empagliflozin

This was assessed by comparing weight, body mass index, random blood sugars and HbA1c between the two groups and compared them before and after fasting:

### Comparison of Weight, BMI, RBS, HbA1c between two groups before and after Ramadan

- ∙ There was improvement in blood sugar levels after use of SGLT-2i before and after Ramadan (FBS 141 ± 49 vs 125 ± 41) and (RBS 181 ± 64 vs 162 ± 53).
- ∙ HbA1c also improved after use of SGLT-2i before and after Ramadan (7.2 ± 0.8 vs 6.9 ± 0.9% for Metformin + Empagliflozin and 7.8 ± 1.5 vs 7.6 ± 1.6% for Metformin and sitagliptin).
- ∙ Weight and BMI improved after use of SGLT-2i (BMI 36.5 ± 4.8 kg/m^2^ before Ramadan and 33.7 ± 2.4 kg/m^2^ after Ramadan) (Tables 3 and 4).
- ∙ None of the studied patients discontinued the prescribed medications.

**Table 3:** Comparison of Metformin + Sitagliptin and Metformin + Empagliflozin pre and post Ramadan fasting

Groups	Variables	Before Ramadan	After Ramadan	*P* value
**Group A (Metformin + Sitagliptin)** ***n* = 88**	Systolic BP (mmHg)	129.55 ± 15.2	128.9 ± 16.4	0.751
	Diastolic BP (mmHg)	81.1 ± 10.4	83.9 ± 14.7	0.124
	Weight (Kg)	77.6 ± 13.7	77.6 ± 4.0	0.987
	BMI (kg/m^2^)	39.5 ± 5.7	34.9 ± 4.8	0.093
	RBS (mg/dl)	209 ± 99	243 ± 44	0.452
	HbA1C (%)	7.8 ± 1.5	7.6 ± 1.6	0.016*
**Group B (Metformin + Empagliflozin) *n* = 44**	Systolic BP (mmHg)	122.8 ± 17.9	123.3 ± 16.4	0.873
	Diastolic BP (mmHg)	81.8 ± 8.7	85.0 ± 18.2	0.178
	Weight (Kg)	85.3 ± 7.8	80.5 ± 8.4	0.716
	BMI (kg/m^2^)	36.5 ± 4.8	33.7 ± 2.4	0.165
	RBS (mg/dl)	181 ± 64	162 ± 53	0.398
	HbA1C (%)	7.2 ± 0.8	6.9 ± 0.9	0.027*

^*^P value calculated using Paired sample *T* test, keeping *P* value ≤ 0.5 as significant

BMI: body mass index, RBS: random blood sugar BP: blood pressure

**Table 4:** Comparison between Group A (Metformin + Sitagliptin) and Group B (Metformin + Empagliflozin)

Variables	Before/After Ramadan	Group A (*n* = 88) (Metformin + Sitagliptin)	Group B (*n* = 44) (Metformin + Empagliflozin)	*P* value
**Age (years) mean ± SD**		50.6 ± 10.5	44.7 ± 10.7	0.027*
**Systolic BP (mmHg)**	Before Ramadan	129.55 ± 15.2	122.8 ± 17.9	0.028*
	After Ramadan	128.9 ± 16.4	123.3 ± 16.4	0.065
**Diastolic BP (mmHg)**	Before Ramadan	81.1 ± 10.4	81.1 ± 8.7	0.711
	After Ramadan	83.9 ± 14.7	85.0 ± 18.2	0.721
**Weight (Kg)**	Before Ramadan	77.6 ± 13.7	85.3 ± 7.8	0.007*
	After Ramadan	77.6 ± 4.0	80.5 ± 8.4	0.002*
**BMI (Kg/m**^**2**^**)**	Before Ramadan	39.5 ± 5.7	36.5 ± 4.8	0.155
	After Ramadan	34.9 ± 4.8	33.7 ± 2.4	0.470
**RBS (mg/dl)**	Before Ramadan	209 ± 99	181 ± 64	0.005*
	After Ramadan	243 ± 44	162 ± 53	0.293
**HbA1C (%)**	Before Ramadan	7.8 ± 1.5	7.2 ± 0.8	0.038*
	After Ramadan	7.6 ± 1.6	6.9 ± 0.9	0.019*

*BMI* Body mass index, *RBS* Random blood sugar, *BP* Blood pressure

^*^P value calculated using Independent sample *T* test, keeping *P* value ≤ 0.5 as significant

The levels of BMI after Ramadan fasting showed a non-significant difference between the SGLT-2 inhibitors group and Sitagliptin group (*P* value 0.15 before Ramadan and *P* = 0.47 after Ramadan).

## Discussion

Sodium glucose co-transporter inhibitors (SGLT2i) is an emerging class of anti-diabetic medications which primarily act by inhibiting SGLT2 receptors located on the proximal glomerular tubules leading to significant glycosuria along with osmotic diuresis. The net consequence of this mechanism is insulin independent lowering of glucose levels in addition to modest weight loss. Owing to its mechanism of action, SGLT2i are generally not associated with hypoglycemia per se; however, it is unclear whether this risk changes during prolonged ritual fasting as observed by Muslims in Ramadan. Furthermore, risk of volume depletion may be especially pronounced during fasting, manifesting in the form of postural hypotension, dehydration, dizziness, and blackout, all of which may lead to falls and other potentially serious medical consequences. Additionally, an increased risk of urinary tract infections and genital infections is recognized with the use of SGLT2i. Finally, SGLT2i are associated with rare but clinically significant complication of euglycemic diabetic ketoacidosis. The later risk is particularly observed in people rapidly lowering or stopping concurrent insulin use. Considering all of the aforementioned knowledge, there is widespread concerns and uncertainty regarding safety of SGLT2i in patients with T2D who practice ritual fasting in Ramadan. This study, therefore, aimed to ascertain safety of SGLT2i during fasting.

In our study, comparable rates of hypoglycemic episodes were observed in both Empagliflozin and Sitagliptin groups (4.16% vs 8.7%, *P* = 0.65). These findings are consistent with another study conducted by Wan Seman WJ et al., which compared SGLT2i with Sulphonylureas in the management of diabetes during Ramadan and observed fewer episodes of symptomatic and severe hypoglycaemia among SGLT2i users compared to Sulphonylureas [12, 13]. Similarly, there was statistically no significant difference in rates of dehydration between groups taking Sitagliptin and Empagliflozin (7.6% vs. 11.1%). A slightly higher reported symptom of dehydration can be explained by the volume lowering properties of SGLT2 inhibitors however it was not significant enough to discontinue medication during fasting. Although patients reported higher urinary frequency with Empagliflozin, there was no observed difference in blood pressure measurement to account for significant volume depletion. It should be noted that blood glucose level for study participants was optimized before Ramadan according to DAR-IDF guidelines and this may account for lower episodes of dehydration which reinforces the importance of attaining optimal glycemic control before Ramadan to reduce the risk of dehydration with SGLT2i [3].

In terms of risk of urinary tract infections and genital infections, our study did not find statistically significant difference between two groups. Similarly, no documented episodes of euglycemic diabetic ketoacidosis were reported in either group. We screened urine for ketones as serum ketone measurement was not available as part of surveillance, no clinical manifestation in the form of diabetic ketoacidosis was observed during study period. In an observational study performed in Singapore, no increase in ketone levels was observed during Ramadan in a cohort using SGLT2i [14]. Nonetheless, risk of diabetic ketoacidosis with SGLT2i is found to be associated with rapid lowering or cessation of exogenous insulin. As it is common practice to adjust and/or lower insulin dose during Ramadan to correspond with altered meal timings and frequency, caution should be exercised with appropriate counseling of the patient when starting SGLT2i while lowering insulin dose.

Both Sitagliptin and Empagliflozin were associated with reduction in HbA1c and random blood glucose values as well as weight and BMI. However, difference of weight between both groups was not observed in this study, possibly because use of SGLT2i was started before initiation of the study. This suggests that Empagliflozin retains its glucose and weight lowering properties without adverse effects in carefully selected patients who choose to fast during Ramadan.

As mentioned above, no serious adverse effects were observed which could lead to discontinuation of SGLT2i in our cohort of participants using it during Ramadan fasting.

Our study has number of limitations which should be taken into account while interpreting findings. As an observational study, it has inherent bias imbedded in studies of similar nature such as recall bias which cannot be excluded in this case as rate of symptomatic hypoglycemia and dehydration was dependent on patients’ reports. Secondly, this was a small sample size study which again limits extrapolation of our findings on larger population groups. [15] Similarly, the 2:1 ratio between participants taking sitagliptin and empagliflozin during fasting must be taken into account when comparing differences as less adverse effects in empagliflozin group may be the consequence of very small cohort size. This ratio of 2:1 was deliberately chosen by investigators owing to baseline concerns of SGLT2i safety during prolonged fasting. This decision was taken with patient safety in mind and to minimize any untoward complications keeping in view that some patients may drop out of the study during Ramadan.

The strength of this study is that it is the first study of its kind to address the safety concerns of SGLT2 inhibitors during fasting in Pakistani population. In addition, we are not aware of any previous studies directly comparing SGLT2i with DPP4 inhibitors in people with T2DM who wish to fast during Ramadan. As an observational study, physicians prescribing either Sitagliptin or Empagliflozin had full professional discretion based on their clinical judgment which is in keeping with usual day to day care thus making our findings relevant to real world practice. A robust clinical follow up and periodic biochemical assessment before, during and after Ramadan was in place with high retention rate of study participants thus lending credibility to our findings.

Various trials performed to demonstrate cardiovascular safety of SGLT2i class demonstrated astonishingly positive outcomes with statistically significant reduction in progression of heart failure and renal dysfunction thus setting their place in the therapeutic management of heart failure and chronic renal disease independent of glucose lower properties. We postulate that our findings add to the evidence and clinical assurance that SGLT2 inhibitors may also be used or continued for their cardio renal protective properties in people without diabetes who wish to fast during Ramadan.

## Conclusion

In people with Type 2 diabetes mellitus fasting during Ramadan, use of SGLT-2 inhibitors combined with metformin is as safe, effective and well-tolerated as DPP4 inhibitors combined with metformin. Both combinations are effective in improving weight and HbA1c. However, DPP4 inhibitors were associated with more adverse effects than SGLT-2 inhibitors.

## Data Availability

The datasets used and/or analysed during the current study are available from the corresponding author on reasonable request.
